# Abnormal phosphorylation of Tie2/Akt/eNOS signaling pathway and decreased number or function of circulating endothelial progenitor cells in prehypertensive premenopausal women with diabetes mellitus

**DOI:** 10.1186/s12902-016-0093-y

**Published:** 2016-03-02

**Authors:** Haitao Zeng, Yanping Jiang, Hailin Tang, Zi Ren, Gaofeng Zeng, Zhen Yang

**Affiliations:** Center for Reproductive Medicine, The Sixth Affiliated Hospital, Sun Yat-sen University, Guangzhou, 510080 People’s Republic of China; Department of Cardiovascular Medicine, The Second Affiliated Hospital of University of South China, Hengyang, Hunan 421001 China; Cancer Center, Sun Yat-sen University, Guangzhou, 510080 People’s Republic of China; Department of Hypertension & Vascular Disease, The First Affiliated Hospital, Sun Yat-Sen University, Guangzhou, 510080 People’s Republic of China

**Keywords:** Prehypertension, Diabetes mellitus, Endothelial progenitor cells, Endothelial function, Tie2 signaling pathway

## Abstract

**Backgrounds:**

The number and activity of circulating endothelial progenitor cells (EPCs) in prehypertension is preserved in premenopausal women. However, whether this favorable effect still exists in prehypertensive premenopausal women with diabetes is not clear.

**Methods:**

This study compared the number and functional activity of circulating EPCs in normotensive or prehypertensive premenopausal women without diabetes mellitus and normotensive or prehypertensive premenopausal women with diabetes mellitus, evaluated the vascular endothelial function in each groups, and investigated the possible underlying mechanism.

**Results:**

We found that compared with normotensive premenopausal women, the number and function of circulating EPCs, as well as endothelial function evaluated by flow-mediated dilatation (FMD) in prehypertensive premenopausal women were preserved. In parallel, the Tie2/Akt/eNOS signaling pathway and the plasma NO level or NO secretion of circulating EPCs in prehypertensive premenopausal women was also retained. However, in presence of normotension or prehypertension with diabetes mellitus, the number or function of circulating EPCs and FMD in premenopausal women decreased. Similarly, the phosphorylation of Tie2/Akt/eNOS signaling pathway and the plasma NO level or NO secretion of circulating EPCs was reduced in prehypertension premenopausal with diabetes mellitus.

**Conclusion:**

The present findings firstly demonstrate that the unfavorable effects of diabetes mellitus on number and activity of circulating EPCs in prehypertension premenopausal women, which is at least partially related to the abnormal phosphorylation of Tie2/Akt/eNOS signaling pathway and subsequently reduced nitric oxide bioavailability. The Tie2/Akt/eNOS signaling pathway may be a potential target of vascular protection in prehypertensive premenopausal women with diabetes mellitus.

## Background

Prehypertension is an intermediate state between normotension and hypertension, which is associated with increased cardiovascular risks [1–6]. Endothelial dysfunction, accompanied by prehypertension, plays a crucial role in initiation and progression of atherosclerotic vascular disease and subsequent clinical consequences [7–9]. Increasing evidence suggests that circulating endothelial progenitor cells (EPCs) are thought to be a subset of cells derived from the bone marrow that plays a pivotal role in accelerating reendothelialization, repairing endothelial injury and improving endothelial function [10–13]. However, in presence of risk factors for cardiovascular disease such as prehypertension, obesity and smoke, the number and activity of circulating EPC decreased, which may contribute to the development of endothelial injury [14–16].

It is well known that diabetes mellitus (DM) is a complex metabolic disorder characterized by increased morbidity and mortality rates. Compared with nondiabetic controls, patients with DM have a high risk of developing cardiovascular complications [17, 18], which correlated with endothelial injury and dysfunction [19–22]. In presence of diabetes, the number and activity of circulating EPCs, as well as flow-mediated dilatation (FMD) were impaired [23–25]. Meanwhile, the number and activity of circulating EPCs is related to endothelial function as evaluated by measurement of FMD [26, 27]. These results implies that the attenuated endothelial repair capacity may be lead to diabetes-related vascular injury.

It has proved that the occurrence of cardiovascular risk is lower in premenopausal women than postmenopausal women and age-matched men [28, 29]. The increased cardiovascular diseases risks in postmenopausal women may be partly associated with endothelial injury and dysfunction [30]. Our previous studies showed that the number and activity of circulating EPCs in prehypertensive premenopausal women were preserved, indicating the retained endothelial repair capacity may contribute to vascular protection concomitant with prehypertensive premenopausal women [31]. However, whether the beneficial effect still exists in prehypertensive premenopausal women with diabetes is still unclear. According to the previous studies revealing the unfavorable effect of DM on circulating EPCs [23–25], we hypothesized that the number and activity of circulating EPCs might be impaired in premenopausal prehypertensive women with diabetes. In addition, nitric oxide (NO), vascular endothelial growth factors (VEGF), and granulocyte macrophage colony- stimulating factor (GM-CSF) regulate the number and function of circulating EPCs [32–35]. EPC-mediated endothelial repair capacity is associated with NO secretion by EPCs [36]. Tie2/PI3K/Akt signaling pathway is one major upstream effectors of eNOS. Our study and other investigations indicated that the Tie2/Akt/eNOS signaling play an important role in regulating the function of circulating EPCs [37–40]. Based on the prior reports, we further assumed that the changes of EPCs in the three groups might be related to the alteration of NO, VEGF, GM-CSF level in plasma or secretion by circulating EPCs, and downregulation of Tie2/Akt/eNOS signaling pathway in circulating EPCs might be also responsible for decreased EPCs functional activity in premenopausal prehypertensive women with diabetes. Hence, we observed normotensive or prehypertensive premenopausal women without diabetes mellitus and normotensive or prehypertensive premenopausal women with diabetes mellitus, evaluated the levels of NO、 VEGF and GM-CSF in plasma or culture medium, and investigated the expression of tie2/Akt/eNOS signaling pathway in each groups. This study may provide useful information on our understanding of the novel mechanism underlying the alteration of endothelial repair capacity in prehypertensive premenopausal women with diabetes mellitus.

## Methods

### Characteristics of subjects

Twenty normotensive premenopausal women, twenty prehypertensive premenopausal women, twenty normotensive premenopausal women with type 2 diabetes mellitus, and 20 prehypertensive premenopausal women with type 2 diabetes mellitus were studied. The enrolled subjects had no known cardiovascular disease or ongoing drug treatments (such as antiplatelet, anti-inflammatory or hypolipidemic agents). The normotensive premenopausal women had no cardiovascular risk factors, a systolic blood pressure < 120 mmHg, and a diastolic blood pressure <80 mmHg. The prehypertensive women were diagnosed as systolic blood pressure between 120 and 139 mmHg or diastolic blood pressure between 80 and 89 mmHg according to the Eight Joint National Committee (JNC 8) guidelines [41]. Prehypertensive women with type 2 diabetes mellitus was newly diagnosed without any drug treatments according to the guidelines in the Expert Committee Report of the American Diabetes Association [42]. The subjects with smokers, malignant disease, infection, or inflammatory disorders were excluded as all these conditions may influence the number or activity of EPC. The subjects with breastfeeding or pregnant women and those with previous hysterectomy or irregular menstrual cycles were also excluded. The protocol of this study was approved by the Ethics Committee of the sixth affiliated hospital of Sun Yat-Sen University, and written informed consent for participation in the study was obtained from participants. The main characteristics of the study population are shown in Table 1.

**Table 1:** Clinical and biochemical characteristics

Characteristics	Normotensive women	Prehypertensive women	Normotensive women with DM	Prehypertensive women with DM
	(*n* = 20)	(*n* = 20)	(*n* = 20)	(*n* = 20)
Age (years)	42.7 ± 5.8	44.9 ± 4.0	45.2 ± 6.6	45.4 ± 5.1
Height (cm)	162.3 ± 5.7	160.5 ± 5.9	161.9 ± 6.1	161.1 ± 5.2
Weight (Kg)	58.7 ± 5.7	59.5 ± 5.7	62.1 ± 4.8	64.1 ± 4.4
BMI (kg/cm^2^)	23.5 ± 2.7	22.9 ± 2.1	24.0 ± 2.1	23.8 ± 1.5
Systolic blood pressure (mmHg)	108.8 ± 6.5	130.4 ± 5.5^a^	111.0 ± 6.0	131.3 ± 4.9^a^
Diastolic blood pressure (mmHg)	68.5 ± 5.0	80.3 ± 4.4^a^	70.9 ± 4.8	80.9 ± 5.4^a^
Heart rate (beats/min)	72.2 ± 7.9	72.8 ± 7.4	73.7 ± 10.2	75.0 ± 7.7
AST (mmol/L)	24.3 ± 5.8	26.9 ± 8.1	27.7 ± 6.0	25.7 ± 6.5
ALT (mmol/L)	22.9 ± 5.2	24.5 ± 5.1	24.8 ± 6.1	21.7 ± 5.7
BUN (mmol/L)	5.7 ± 0.8	5.5 ± 1.2	5.9 ± 1.3	5.4 ± 1.1
Cr (mmol/L)	72.2 ± 14.8	66.4 ± 16.4	73.1 ± 15.2	65.2 ± 18.0
LDL (mmol/L)	2.98 ± 0.33	2.88 ± 0.55	3.10 ± 0.37	3.09 ± 0.36
TC (mmol/L)	4.93 ± 0.44	4.92 ± 0.65	5.11 ± 0.39	5.08 ± 0.42
HDL (mmol/L)	1.37 ± 0.24	1.40 ± 0.21	1.31 ± 0.19	1.34 ± 0.16
TG (mmol/L)	1.44 ± 0.19	1.39 ± 0.17	1.50 ± 0.21	1.46 ± 0.12
FPG (mmol/L)	4.71 ± 0.67	4.51 ± 0.61	8.91 ± 1.16^b^	8.73 ± 1.08^b^
2hPG(mmol/L)	6.25 ± 0.66	6.17 ± 0.67	10.87 ± 1.72^b^	10.31 ± 1.61^b^
HbA_1_C(%)	5.31 ± 0.58	5.18 ± 0.63	8.49 ± 1.47^b^	8.15 ± 1.52^b^
hrCRP (mmol/L)	0.961 ± 0.509	1.130 ± 0.615	1.953 ± 0.852^b^	1.828 ± 0.882^b^
FMD(%)	10.29 ± 1.16	9.79 ± 1.22	5.87 ± 5.49^b^	5.49 ± 0.129^b^
GMD(%)	22.49 ± 2.12	21.89 ± 2.45	21.05 ± 2.47	21.37 ± 2.68

*Abbreviation*: *BMI* body mass index, *LDL* low-density lipoprotein, *TC* total cholesterol, *HDL* high density lipoprotein, *TG* triglyeride, *FPG* fasting plasma glucose, *2hPG* 2-h plasma glucose, *HbA*_*1*_ *C* glycosylated hemoglobin, *hrCRP* hyper-sensitive C-reactive protein, *GMD* glyceryl trinitrate-mediated dilation, *FMD* flow-mediated brachial artery dilatation

*Notes*: Data are given as mean ± SD. ^a^vs normotension; ^b^vs without DM

All peripheral venous blood samples used for EPCs isolation and routine blood and biochemistry tests including serum total cholesterol, triglycerides, high-density lipoprotein cholesterol, LDL-cholesterol, fasting blood glucose (FBG) and high-sensitivity CRP were taken from the three groups in the morning after overnight fasting. All subjects received checks, menstrual periods of the menstrual cycle (day 2 to day 5 after the first day of menstrual bleeding).

### Evaluation of number of circulating EPCs by flow cytometry analysis and cell culture assay

Detection of EPCs was performed as our previous studies and other investigation described [40, 43]. The count of circulating EPCs was evaluated by the ratio of CD34 + KDR+ cells per 100 peripheral blood mononuclear cells (PBMNCs). The circulating EPCs were isolated, cultured and quantified using DiI-acLDL uptake and FITC-labled Ulex europeus agglutin (lectin) staining as our previous described report [40]. The cultured EPC number was evaluated by DiI-acLDL/lectin doublepositive cells/×200, and counted manually by two independent observers blinded to the study.

### Migration and Proliferation assay of circulating EPCs

EPC migration and proliferation assay was determined as our previous studies reported [40, 43]. EPCs were harvested by centrifugation, resuspended in 500 μl EBM, and counted. Then, 2 × 10^4^ EPCs were placed in the upper chamber of a modified Boyden chamber. The chamber was placed in a 24-well culture dish containing EBM and human recombinant VEGF (50 ng/mL). After incubation at 37 °C for 24 h, the lower side of the filter was washed with PBS and fixed with 2 % paraformaldeyde. For quantification, cell nuclei were stained with DAPI. Cells migrating into the lower chamber were counted manually in 3 random microscopic fields.

EPC proliferation was performed as following. After being cultured for 7 days, EPCs were digested with 0.25 % trypsin and then cultured in serum-free medium in 96-well culture plate (200 μL/well). After being cultured for 24 h, EPCs were supplemen-ted with 10 μ l MTT (5 g/L, Fluka Co. Product) and incubated for another 4 h. Then the supernatant was discarded by aspiration and the EPC preparation was shaked with 200 μ L dimethyl sulfoxide (DMSO) for 10 min, before the OD value was measured at 490 nm.

### Measurement of NO、VEGF and GM-CSF levels in plasma and secretion by EPCs

Plasma was assayed for NO、VEGF and GM-CSF levels. Nitrite, the stable metabolite of nitric oxide, was estimated by using Greiss method as our previous studies reported [32]. The formation of nitrite (NO_2_^−^) and nitrate (NO_3_^−^) was detected in cell culture supernatants. This assay determined the total NO based on the enzymatic conversion of NO_3_^−^ to NO_2_^−^ by nitrate reductase (Sigma), and detection of nitrite as an azo dye product of the Greiss reaction. The results are presented as μmol NO_x_ of NO_3_ˉ/NO_2_^−^ per liter of medium. Plasma levels of VEGF and GM-CSF were measured by high-sensitive ELISA assays (R&D Systems, Wiesbaden, Germany) according to our previous studies [32].

The cultured EPCs were switched to the DMEM/20 %-fetal bovine serum (no supplemental growth factors) for 48 h. Then, the conditioned media were assayed for NO、VEGF and GM-CSF as previously described [32].

### Flow mediated dilatation

The measurement of FMD were performed according to the previous reports [44, 45]. Brachial artery FMD was measured by highresolution ultrasonography using a 5–12 MHz linear transducer on an HDI 5000 system (Washington, USA). The brachial artery was studied 20 to 100 mm proximal to the antecubital fossa in supine participants after 15 min rest [44, 45]. Pressure in an upper-forearm sphygmomanometer cuff was raised to 250 mmHg for 5 min and FMD calculated as the percentage increase in mean diastolic diameter after reactive hyperaemia 55 to 65 s after deflation to baseline [44, 45]. After a further 15 min, 400 μg sublingual glyceryl trinitrate (GTN) was given and diastolic diameter remeasured after 5 min for measurement of endothelial-independent dilatation [44, 45].

### Western blot analysis

EPC proteins were harvested by cell lysis buffer (Cell SignalingTechnology Inc, Danvers, MA, USA). Protein extracts were subjected to SDS-PAGE, transferred to polyvinylidene fluoride membranes(Cell Signaling Technology Inc.). The following antibodies were used: rabbit phospho-Tie2, Tie2, phospho-Akt, Akt, phospho-eNOS, eNOS(1:1000; Cell Signaling Technology Inc.) andβ-actin (1:1000; Santa Cruz Technology Inc.). Proteins were visualized with HRP-conjugated anti-rabbit IgG (1:2000; Cell Signaling Technology Inc.) or HRP-conjugated anti-mouse IgG (1:5000; Cell Signaling Technology Inc.), followed by use of the ECL chemiluminescence system (Cell Signaling Technology Inc.). The intensity of immunoreactive bands was analyzed. The results were expressed as the ratio of specific phosphorylated proteins to total proteins for the phosphorylation of Tie2, Akt, and eNOS in human EPCs, and the ratio of total Tie2, Akt, and eNOS proteins to ß-actin for expression of Tie2, Akt, and eNOS in human EPCs. The statistical comparisons for western blot analysis were made relative to normotension.

### Statistic analysis

The statistic software was SPSS V11.0 (SPSS Inc., Chicago, Illinois). All data were expressed as mean ± SD. Comparisons between the four groups were analyzed by two-factor analysis of variance (status of with or without diabetes mellitus, status of normotension or prehypertension). When indicated by a significant *F*-value, a post hoc test using the Newman-Keuls method identified significant differences among mean values. Comparisons in Tie2/Akt/eNOS signaling pathway between the three groups were analyzed by using the unpaired Student’s *t* test and one-way ANOVA. Univariate correlations were calculated using Pearson’s coefficient (r). *P*-values less than 0.05 were considered statistical significance.

## Results

### Clinical characteristics

As shown in Table 1, the four groups were similar in terms of age and BMI. There were no differences between the levels of triglycerides, cholesterol, LDL-cholesterol, high-density lipoprotein cholesterol in the three groups (*P* > 0.05). Compared with normotensive premenopausal women with or without diabetes mellitus, the systolic and diastolic blood pressure in prehypertensive premenopausal women with or without diabetes mellitus was higher (*P* < 0.05). FBG, 2hPG, HbA1C, FMD and hr-CRP in normotensive or prehypertensive women with diabetes mellitus were significantly higher than those in normotensive or prehypertensive women without diabetes mellitus (*P* < 0.05). However, there were no differences in FMD between normotensive and prehypertensive women (*P* > 0.05). The GMD in the four groups was similar (*P* > 0.05).

### The number and function of circulating EPCs in the four groups

**Fig. 1:**
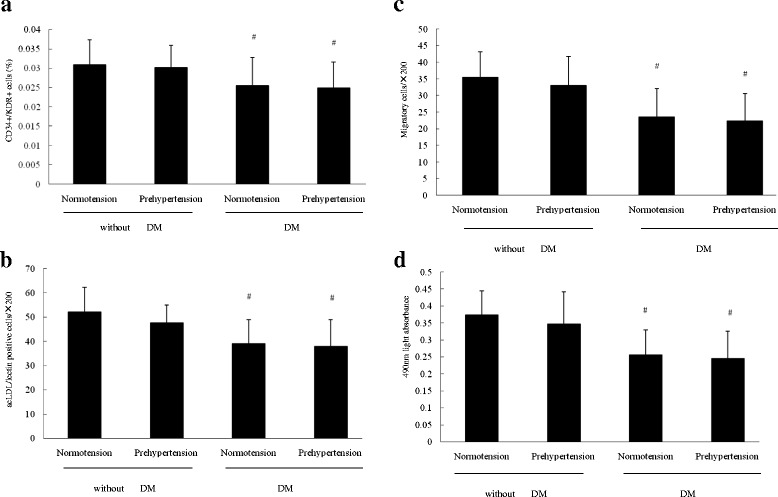
The number and activity of circulating EPCs in the four groups. Evaluated by **a** FACS analysis or **b** cell culture assay, the number of circulating EPCs in prehypertensive premenopausal women without diabetes mellitus was similar to that in normotensive premenopausal women without diabetes mellitus, but higher than that in normotensive or prehypertensive women with diabetes mellitus. There was no difference in the migratory **c** and proliferative **d** activities between normotensive and prehypertensive premenopausal women. The migratory **c** and proliferative **d** activities of cultured EPCs was lower in normotensive or prehypertensive premenopausal women with diabetes mellitus than that in normotensive or prehypertensive premenopausal women without diabetes mellitus. Data are given as mean ± SD (^*^vs normotension; ^#^ vs without DM, *n* = 20 for each group)

As shown in Fig. 1a and b, there was no difference in number of circulating EPCs evaluated by FACS analysis or cell culture assay between normotensive and prehypertensive premenopausal women (*P* > 0.05). However, the number of circulating EPCs was significantly lower in normotensive or prehypertensive premenopausal women with diabetes mellitus than that in normotensive or prehypertensive premenopausal women without diabetes (*P* < 0.05). Figure 1c and d showed that the migratory or proliferative function of EPCs in normotensive premenopausal women was equal to that in prehypertensive premenopausal women (*P* > 0.05). However, the migratory or proliferative function of EPCs in normotensive or prehypertensive premenopausal women with diabetes were lower than those in normotensive or prehypertensive premenopausal women without diabetes (*P* < 0.05). These results indicated that the preserved number and activity of circulating EPCs in prehypertensive premenopausal women was impaired in presence of diabetes.

### Plasma NO、VEGF and GM-CSF levels in the four groups

**Fig. 2:**
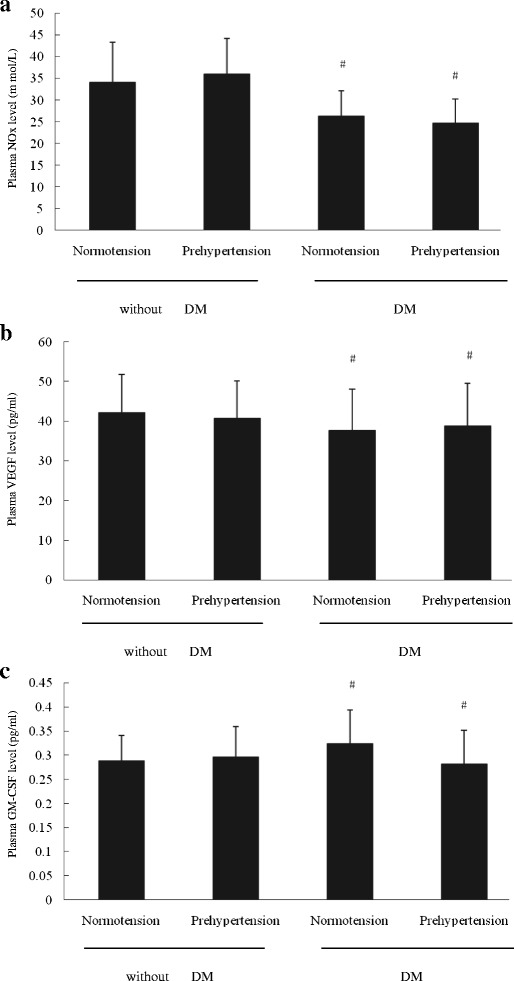
The plasma NO、VEGF and GM-CSF levels in the four groups. **a** The plasma NO level in normotensive premenopausal women without diabetes mellitus was almost equal to that prehypertensive premenopausal women without diabetes mellitus. Compared with normotensive or prehypertensive premenopausal women without diabetes mellitus, the plasma NO level in normotensive or prehypertensive women with diabetes mellitus decreased. No significant difference was found in plasma VEGF **b** or GM-CSF **c** level among the four groups. Data are given as mean ± SD (^*^vs normotension; ^#^ vs without DM, *n* = 20 for each group)

As shown in Fig. 2, the plasma NO levels in normotensive premenopausal women was similar to that in prehypertensive premenopausal women (*P* > 0.05). However, the plasma NO levels in normotensive or prehypertensive premenopausal women with diabetes were significantly lower than those in normotensive or prehypertensive premenopausal women without diabetes (*P* < 0.05). Different from the plasma NO level, there was no significant difference in the plasma VEGF and GM-CSF level among the four groups (*P* > 0.05).

### NO、VEGF and GM-CSF secretion by EPCs in the four groups

**Fig. 3:**
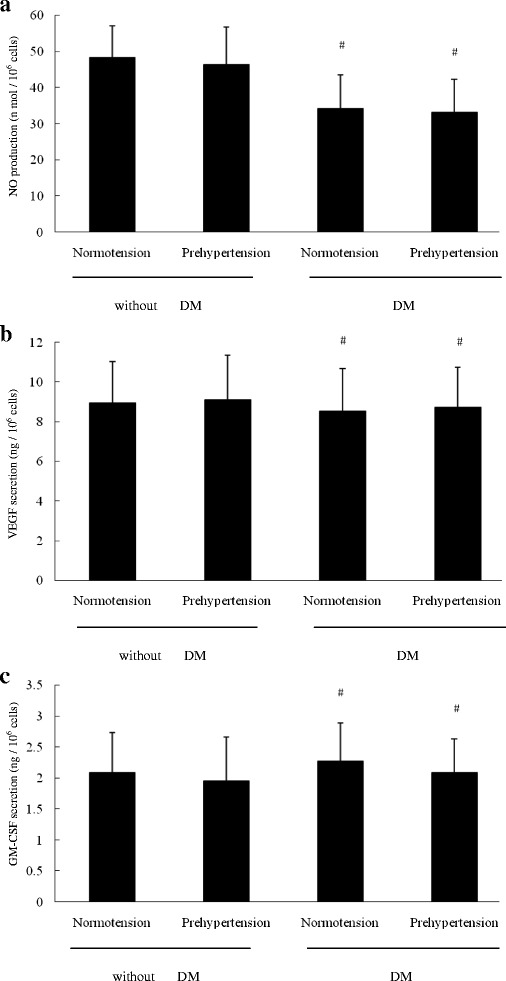
The NO、VEGF and GM-CSF secretion by EPCs in the four groups. **a** The NO secretion by EPCs was the same in normotensive premenopausal women without diabetes mellitus as that in prehypertensive premenopausal women without diabetes mellitus, but higher than that in normotensive or prehypertensive women with diabetes mellitus. There was no difference in VEGF **b** or GM-CSF **c** secretion by EPCs among the four groups. Data are given as mean ± SD (^*^vs normotension; ^#^ vs without DM, *n* = 20 for each group)

Figure 3 showed the NO、VEGF and GM-CSF secretion by EPCs in the four groups. The NO secretion by cultured EPCs in normotensive premenopausal women without diabetes was almost equal to that in prehypertensive premenopausal women without diabetes (*P* > 0.05), but higher than that in normotensive or prehypertensive women with diabetes mellitus (*P* < 0.05). However, no difference was shown among the four groups in term of either VEGF or GM-CSF secretion by cultured EPCs (*P* > 0.05).

### Correlation between circulating EPCs or NO level and FMD

**Fig. 4:**
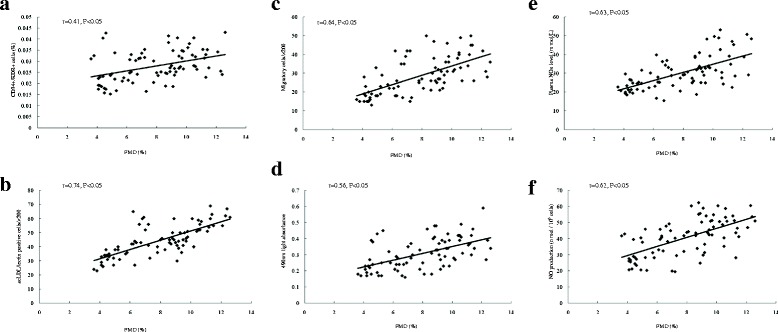
The correlation between circulating EPCs or NO level and FMD. **a** and **d** showed that the correlation between the number or activity of circulating EPCs and FMD. The number of circulating EPCs evaluated by FACS **a** or by cell culture **b** strongly correlated with the FMD. There was a correlation between the EPC proliferatory **c** or migratory **d** and FMD. **e** and **f** showed that the correlation between the plasma NO level and NO secretion by EPCs and FMD. There was a correlation between the plasma NO level **e** or NO secretion by EPCs **f** and FMD

Figure 4a and d showed that the correlation between the number or function of circulating EPCs and FMD. There was significant correlation between FMD and the number of circulating EPCs evaluated by flow cytometry analysis (*r* = 0.41, *P* < 0.05) or by cell culture (*r* = 0.74, *P* < 0.05). Similarly, the EPC migration and EPC proliferation significantly correlated with plasma FMD (*r* = 0.64, *P* < 0.05, and *r* = 0.56, *P* < 0.05, respectively). Figure 4e and f showed that the correlation between the plasma NO level and NO secretion by EPCs and FMD. There was obvious correlation between FMD and plasma NO level (*r* = 0.63, *P* < 0.05), and NO secretion by EPCs (*r* = 0.62, *P* < 0.05).

### The Tie2/Akt/eNOS signaling pathway of circulating EPCs in the three groups

**Fig. 5:**
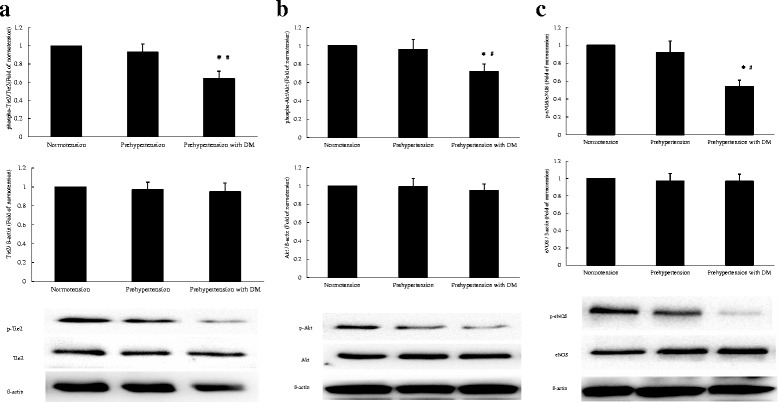
The Tie2/Akt/eNOS signaling pathway of circulating EPCs in the three groups. There was no difference in the phosphorylation of tie2 **a**, Akt **b** and eNOS **c** of circulating EPCs between prehypertensive and normotensive premenopausal women without diabetes mellitus. The phosphorylation of tie2 **a**, Akt **b** and eNOS **c** of circulating EPCs in prehypertensive and normotensive premenopausal women without diabetes mellitus exceeded that in prehypertensive premenopausal women with diabetes. The expression of tie2 **a**, Akt **b** and eNOS **c** of circulating EPCs in the three groups exhibited no difference. Data are given as mean ± SD. (*P < 0.05 compared with normotension without diabetes mellitus, ^#^ *P* < 0.05 compared with prehypertension without diabetes mellitus, *n* = 20 for each group)

To investigate the possible mechanism underlying the deficiency in NO secretion by cultured EPCs in presence of prehypertension with diabetes mellitus, we further evaluated the expression of Tie2/Akt/eNOS signaling pathway of circulating EPCs in the three groups. As shown in Fig. 5, the phosphorylation of tie2, Akt and eNOS of circulating EPCs in prehypertensive premenopausal women and normotensive premenopausal women without diabetes exhibited no significant difference (*P* > 0.05). However, the phosphorylation of tie2, Akt, and eNOS of circulating EPCs in prehypertensive and normotensive premenopausal women without diabetes were higher than that in prehypertensive premenopausal women with diabetes mellitus (*P* < 0.05). In addition, there was no difference in the expression of total tie2, Akt and eNOS of circulating EPCs in the three groups (*P* > 0.05).

## Discussion

The present results indicates that in presence of diabetes mellitus, the preserved endothelial function in prehypertensive premenopausal women was attenuated, which may be related to impaired number and function of circulating EPCs. This alteration in circulating EPCs may be due to decreased NO production, and the abnormal phosphorylation of Tie2/Akt/eNOS signaling pathway may be the underlying mechanism. Therefore, this study firstly demonstrates the unfavorable effects of diabetes mellitus on endothelial function and circulating EPCs in prehypertensive premenopausal women and its possible underlying mechanism.

Diabetes mellitus is a major risk factor for the development of cardiovascular disease and is associated with a high cardiovascular morbidity and mortality [17, 18]. It is generally accepted that the number and function activity of circulating EPCs is impaired in diabetes patients [23–25], supporting the role of suppressed endothelial repair capacity in pathogenesis of diabetes-related vascular abnormalities. Our previous studies show that in prehypertension premenopausal women the number and activity of circulating EPCs were preserved [31], indicating that EPC-mediated vascular protection in prehypertension premenopausal women may due to increased endothelial repair capacity. However, the effect of diabetes on number and activity of circulating EPCs in prehypertension premenopausal women is unclear. In this investigation, we found that in presence of diabetes mellitus, both the number and functional activity of circulating EPCs is impaired in prehypertensive premenopausal women. FMD is regarded as a reliable and reproducible technique for assessment of endothelial dysfunction in cardiovascular disease [44–46]. Our results also revealed that FMD was reduced in prehypertensive premenopausal women in presence of diabetes mellitus and there is a significant correlation between the number or function activity of circulating EPCs and FMD, indicating that the attenuated endothelial repair capacity may lead to endothelial dysfunction in prehypertensive premenopausal women with diabetes mellitus.

NO、VEGF and GM-CSF are the pivotal factors to regulate the number and function of circulating EPCs [32–35]. Previous studies demonstrated that diabetes mellitus impairs eNOS activity, resulting in decreased NO bioavailability [47]. Accordingly, we assumed that the effect of diabetes mellitus on number and activity of circulating EPCs in prehypertension premenopausal women might be associated with NO、VEGF and GM-CSF. In result, we found that compared with normotensive premenopausal women, the plasma NO level or NO secretion by EPCs was restored in prehypertension premenopausal women, but reduced in prehypertensive premenopausal women with diabetes mellitus. The present results indicates that diabetes-related decreased number and activity of circulating EPCs in prehypertension premenopausal women may be related to the reduced NO production. However, no difference in VEGF or GM-CSF in plasma or secretion by cultured EPCs was found among the three groups, suggesting that the alteration of endothelial repair capacity in prehypertensive premenopausal women with diabetes mellitus may be independent of the changes in plasma VEGF or GM-CSF level or secretion by cultured EPCs. In addition, we also found that there was a positive correlation between the plasma NO level or NO secretion by EPCs and FMD, further supporting that both reduced systemic NO production and endogenous NO biosynthase by circulating EPCs may contribute to endothelial dysfunction in prehypertensive premenopausal women with diabetes mellitus.

Tie2/Akt/eNOS pathway plays an important role in regulating the number and function of circulating EPCs and subsequently accelerating endothelial repair capacity for vascular injury [48–51]. In this investigation, we found that when compared with normotensive premenopausal women, the phosphorylation of tie2, Akt, and eNOS of circulating EPCs were preserved in prehypertensive premenopausal women without diabetes, but reduced in prehypertensive premenopausal women with diabetes mellitus. However, the expression of tie2, Akt and eNOS of circulating EPCs showed no difference in the three groups. These results indicate that the decreased phosphorylation of tie2, Akt, and eNOS but not the alteration of expression of Tie2/Akt/eNOS pathway contributes to reduced NO secretion by circulating EPCs in prehypertensive premenopausal women with diabetes mellitus. Interestingly, the phosphorylation of eNOS was slightly lower than the phosphorylation of tie2 and Akt of circulating EPCs in prehypertensive premenopausal women with diabetes mellitus, implying that other mechanism may be involved in abnormal activity of eNOS of circulating EPCs. Previous study reported that in circulating EPCs in presence of type 2 diabetes, GTPCH Ι deactivation leads to BH4 efficiency and subsequent eNOS uncoupling [52]. Thus, we inferred that GTPCH Ι deactivation may be another mechanism underlying the decreased phosphorylation of eNOS of circulating EPCs in prehypertensive premenopausal women with diabetes mellitus.

The present study has some implications as follows. First, different from prehypertensive premenopausal women without diabetes mellitus, the endothelial function together with number or activity of circulating EPCs in prehypertensive premenopausal women with diabetes mellitus were impaired, indicating that the intervention to improve endothelial function and vascular repair capacity may be necessary for prehypertensive premenopausal women with diabetes mellitus. Second, our results show that diabetes mellitus impaired the number or function of circulating EPCs and subsequently reduced NO production in prehypertensive premenopausal women, which is at least in part mediated by tie2/Akt/eNOS signaling pathway. The date reported here proved that in presence of prehypertension with diabetes mellitus, the tie2/Akt/eNOS signaling pathway in circulating EPCs may be useful for therapeutic approach of endothelial repair capacity.

## Conclusions

The present study for the first time demonstrates the unfavorable effects of diabetes on number and activity of circulating EPCs and endothelial function in prehypertensive premenopausal women, which is associated with decreased NO production and abnormal phosphorylation of Tie2/Akt/eNOS signaling pathway. The attenuated endogenous endothelial repair capacity may be the important mechanism underlying vascular injury in prehypertensive premenopausal women with diabetes. Our findings provide new insight into vascular protection in patients of prehypertensive premenopausal women with diabetes mellitus, suggesting that Tie2/Akt/eNOS signaling pathway may be a potential target for enhancing endogenous endothelial repair capacity.

## Abbreviations

EPCs: Endothelial progenitor cells
DM: Diabetes mellitus
FMD: Flow-mediated dilatation
NO: Nitric oxide
GM-CSF: Granulocyte macrophage colony-stimulating factor
VEGF: Vascular endothelial growth factors
Tie-2: Tyrosine kinase with immunoglobul in and epidermal growth factor homology domain-2
Akt: Protein kinase B
eNOS: Endothelial nitric oxide synthase
GMD: Glyceryltrinitrate-mediated dilation
MTT: 3-(4,5-Dimethylthiazol-2-yl)-2,5-Diphenyltetrazolium bromide
SD: Standard deviation
ANOVA: Analysis of variance
FACS: Fluorescence-activated cell sorting
NOx: Nitrate plus nitrite
GTPCH: Guanosine triphosphate cyclohydrolase
BH4: Tetrahydrobiopterin
